# Supplementary material for: Empirical methods for controlling false positives and estimating confidence in ChIP-Seq peaks
Source: BMC Bioinformatics. 2008 Dec 5;9:523. doi: 10.1186/1471-2105-9-523 (PMC2628906; doi:10.1186/1471-2105-9-523)
Supplement: Additional File 1 — A variety of html documents from the USeq web site detailing the available applications, their best usage, output file type descriptions, command line menus, etc. [file 1471-2105-9-523-S1.zip › USeqUserGuides/JavaDoc.zip!/JavaDoc/bioroot/antibody/UseDilution.html]

UseDilution


|  |  |  |
| --- | --- | --- |
| **Overview** \| **Package** \| **Class** \| **Use** \| **Tree** \| **Deprecated** \| **Index** \| **Help** | |  |
| **PREV CLASS**   NEXT CLASS | **FRAMES**    **NO FRAMES**     **All Classes** |  |
| SUMMARY: NESTED \| FIELD \| CONSTR \| METHOD | DETAIL: FIELD \| CONSTR \| METHOD |  |


---


## bioroot.antibody Class UseDilution

```
java.lang.Object
  bioroot.antibody.UseDilution
```

---

``` public class UseDilution extends java.lang.Object ```

**Author:**
:   nix
    Container to link an antibody use (Western, IP, Immuno, Insitu, etc) to a particular dilution, with notes

---

**Constructor Summary**

`UseDilution()`

`UseDilution(java.sql.ResultSet results,
int antibodyPrepId)`


| **Method Summary** | |
| --- | --- |
| `java.lang.String` | `getAntibodyUse()` |
| `java.lang.String` | `getDilution()` |
| `int` | `getId()` |
| `java.lang.String` | `getNotes()` |
| `void` | `loadUseDilution(java.sql.ResultSet results)` |
| `void` | `setAntibodyUse(java.lang.String antibodyUse)` |
| `void` | `setDilution(java.lang.String dilution)` |
| `void` | `setId(int id)` |
| `void` | `setNotes(java.lang.String notes)` |
| `boolean` | `submitNew(DBUtil bioRoot, int antibodyPrepId)` |
| `boolean` | `updateOld(DBUtil bioRoot)` |

**Methods inherited from class java.lang.Object**

`equals, getClass, hashCode, notify, notifyAll, toString, wait, wait, wait`

**Constructor Detail**

### UseDilution

```
public UseDilution(java.sql.ResultSet results,
                   int antibodyPrepId)
```

---


### UseDilution

```
public UseDilution()
```


**Method Detail**

### loadUseDilution

```
public void loadUseDilution(java.sql.ResultSet results)
```

---


### updateOld

```
public boolean updateOld(DBUtil bioRoot)
```

---


### submitNew

```
public boolean submitNew(DBUtil bioRoot,
                         int antibodyPrepId)
```

---


### getId

```
public int getId()
```

---


### setId

```
public void setId(int id)
```

---


### getNotes

```
public java.lang.String getNotes()
```

---


### setNotes

```
public void setNotes(java.lang.String notes)
```

---


### getAntibodyUse

```
public java.lang.String getAntibodyUse()
```

---


### setAntibodyUse

```
public void setAntibodyUse(java.lang.String antibodyUse)
```

---


### getDilution

```
public java.lang.String getDilution()
```

---


### setDilution

```
public void setDilution(java.lang.String dilution)
```


---


|  |  |  |
| --- | --- | --- |
| **Overview** \| **Package** \| **Class** \| **Use** \| **Tree** \| **Deprecated** \| **Index** \| **Help** | |  |
| **PREV CLASS**   NEXT CLASS | **FRAMES**    **NO FRAMES**     **All Classes** |  |
| SUMMARY: NESTED \| FIELD \| CONSTR \| METHOD | DETAIL: FIELD \| CONSTR \| METHOD |  |


---
